# Spray-Drying Microencapsulation of Natural Bioactives: Advances in Sustainable Wall Materials

**DOI:** 10.3390/ph18070963

**Published:** 2025-06-26

**Authors:** Lauryna Pudžiuvelytė, Eglė Petrauskaitė, Jolita Stabrauskienė, Jurga Bernatonienė

**Affiliations:** 1 Department of Drug Technology and Social Pharmacy, Lithuanian University of Health Sciences, LT-50161 Kaunas, Lithuania; jolita.stabrauskiene@lsmu.lt (J.S.); jurga.bernatoniene@lsmu.lt (J.B.); 2 Institute of Pharmaceutical Technologies, Lithuanian University of Health Sciences, LT-50161 Kaunas, Lithuania; egle.petrauskaite@stud.lsmu.lt

**Keywords:** spray-drying, microencapsulation, bioactive compounds, essential oils, vitamins, polyphenols

## Abstract

**Background/Objectives**: In recent years, increasing attention has been paid to the stabilization of natural biologically active compounds in order to expand their application in the food, pharmaceutical, and cosmetic industries. Such compounds, such as polyphenols, essential fatty acids, or vitamins, are extremely sensitive to environmental factors. This study aims to review the spray-drying-based microencapsulation technology and its application for stabilizing sensitive biologically active substances. **Methods**: This article systematically analyzes the main steps of the spray-drying microencapsulation process and discusses traditional and innovative wall materials, including natural polymers (polysaccharides and proteins), as well as new raw material sources (e.g., yeast cells, canola and pea protein isolates, and hemicelluloses). It also examines the potential of these systems for the stimulated release of active ingredients. **Results**: This review provides a comprehensive overview of the main stages of the spray-drying process and critically examines both conventional (e.g., maltodextrin and gum Arabic) and innovative wall materials (e.g., plant-based proteins and food industry by-products). Studies show that using different wall materials can achieve high encapsulation efficiency, improve the stability of biologically active substances, and control their release. Various compounds have been successfully microencapsulated—polyphenols, essential oils, carotenoids, fatty acids, and vitamins—protecting them from oxidation, light, and temperature. The review identifies key factors that can enhance product quality, increase encapsulation yield, and reduce processing costs and energy input—offering meaningful insights for optimizing the microencapsulation process. **Conclusions**: Spray-drying-based microencapsulation is an advanced technology that effectively protects sensitive active ingredients and allows for wider industrial food, pharmaceutical, and cosmetic applications. In the future, more attention is expected to be paid to personalized formulations, stimulated release systems, and sustainable wall materials from by-products.

## 1. Introduction

In recent years, the growing interest in natural bioactive compounds and functional products has led to intensive innovation to ensure the stability and efficacy of these materials. Many bioactive components, such as polyphenols, essential fatty acids, or vitamins, are highly valued due to their antioxidant, anti-inflammatory, immunomodulatory, and cardioprotective properties. These compounds play a vital role in both the food industry—as natural preservatives, functional additives, or health-enhancing ingredients—and the pharmaceutical field, where they are incorporated into formulations to prevent or manage chronic diseases such as cardiovascular disorders, neurodegenerative conditions, and metabolic syndromes. The global market for natural bioactive ingredients is projected to reach over USD 317 billion by 2030, with annual growth rates exceeding 6–7%, particularly in the sectors of nutraceuticals, functional foods, and personalized medicine [1,2]. However, natural bioactive compounds are highly sensitive to environmental factors—light, oxygen, temperature, and humidity. The main negative impact of these conditions can rapidly degrade their structure and function, causing reduced bioactivity and shelf life. Stabilizing these ingredients is essential to ensure their effectiveness and reliability in consumer products.

One of the most effective solutions to address these challenges is microencapsulation by spray-drying. This method allows for the conversion of liquid material into dry microparticles in a short time and under controlled conditions, thus preserving the functional properties of the bioactive components. Spray-drying offers many advantages over other microencapsulation methods such as freeze-drying, coacervation due to its speed, lower operational costs, suitability for both heat-sensitive and heat-resistant materials, flexibility in capacity design, as well as the ability to control particle size [3,4]. This article examines the main steps of the spray-drying microencapsulation process, discusses traditional and innovative wall materials, and provides examples of how microencapsulation is applied to ensure the stability of various bioactive compounds, such as phytochemicals, essential oils, and vitamins. The review highlights new trends—the use of sustainable encapsulating agents derived from food and agricultural waste and the development of stimulated release systems—which open up new possibilities for the natural, eco-friendly applications in various industries.

## 2. Mechanism of Microencapsulation by Spray-Drying

**Figure 1:**
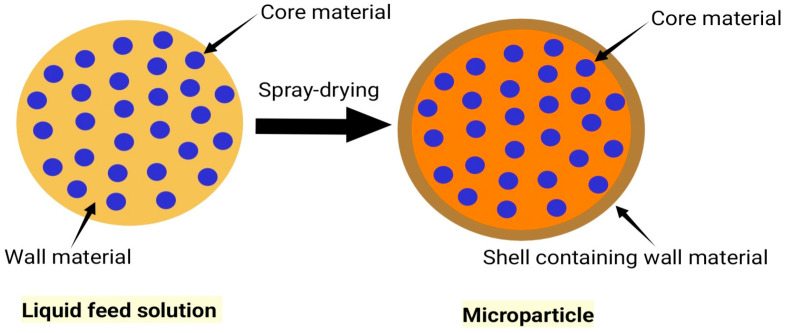
Shell formation around particles during spray-drying.

**Figure 2:**
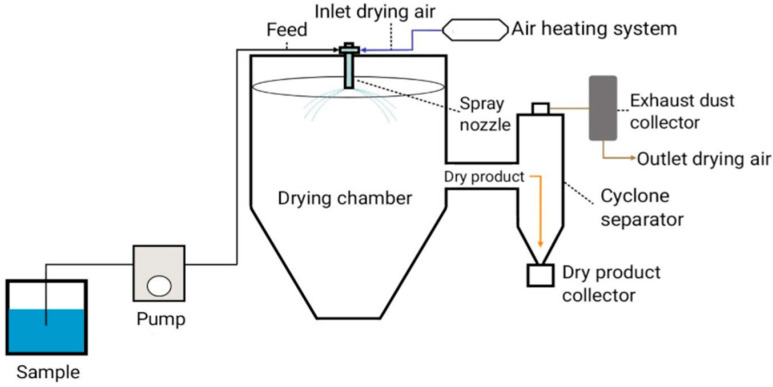
Schematic view of the spray-drying process.

Spray-drying is a widely used encapsulation technique in the pharmaceutical industry due to its simplicity and cost-effectiveness [5], as well as the stability of the encapsulated products [6]. Spray-drying microencapsulation technology relies on forming a membrane/shell around a core material, which is the active ingredient being encapsulated (Figure 1). Spray-drying is suitable for both heat-sensitive and heat-resistant compounds [7]. The principle of the spray-drying method is to form a dry powder from a liquid material by using hot gas [6]. The process involves four fundamental stages: liquid feedstock preparation, the atomization of the feed solution into fine droplets, the drying of the atomized droplets in a hot gas, and, lastly, particle collection (Figure 2) [3]. Some authors use three or five steps to describe this process [5,8].

### 2.1. Liquid Feedstock Preparation

The liquid feed can either be a solution, emulsion, or suspension and should have an optimized viscosity suitable for pumping. Viscous fluids of up to 700 centipoise can typically be sprayed using pressure nozzles, while fluids exceeding 700 centipoise are usually atomized using air atomizing nozzles. The feedstock composition can vary depending on the nature of the active substance and desired product characteristics [3]. To create the liquid feed, core compounds (for example, essential oils (thyme, peppermint, citrus, etc.), polyphenols (rosmarinic acid, resveratrol, caffeinic acid, etc.), vitamins (B1, B6, B12, E, A, D, etc.), and others) are mixed with a wall-forming material (for example, maltodextrin, gum Arabic, sodium caseinate, etc.) solution. For emulsions and suspensions, homogeneity is an important factor affecting the stability of the spray-dried product [6]. During feed preparation, it is crucial to ensure a homogeneous emulsion or suspension and proper viscosity and stability—this helps avoid phase separation and ensures uniform particle formation. The choice of wall and core materials, their concentration, and total solid content have a direct impact on encapsulation efficiency and the powder’s properties.

### 2.2. Atomization

**Figure 3:**
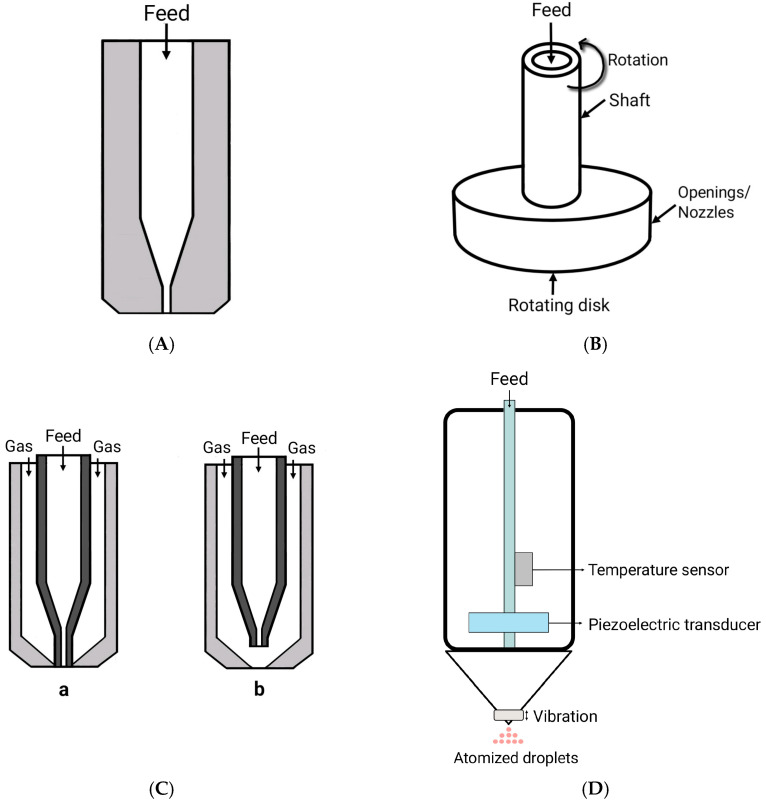
Schematic representation of different types of atomizers used in spray-drying for microencapsulation. (**A**) Pressure atomizer; (**B**) rotary atomizer; (**C**) two-fluid atomizers: a—internal mixing and b—external mixing; (**D**) ultrasonic atomizer.

In this stage, the fluid feed is converted into small droplets through a nozzle [6]. Different types of atomizers (Figure 3A–D) can be used in spray-drying operations [5] according to the desired product characteristics (such as size and shape) and properties of the feed solution [6,8,9]. The nozzle type, feed flow rate, and atomizing air pressure determine droplet size distribution, which directly impacts the drying behavior and final particle morphology. The mean particle size of microcapsules obtained by spray-drying is in the range of 1–100 μm, depending on the nozzle type, capacity, and spraying pressure [10]. Two-fluid spray nozzles are the commonly used atomization devices in the pharmaceutical industry due to their ease of use and ability to handle diverse feedstock materials [3]. Two-fluid nozzles use the pressure of the compressed air (sometimes compressed), and their formatting does not need to be declared.

### 2.3. Drying

This step involves the removal of the solvent from the generated droplets that were produced during atomization. The droplets are introduced into the drying chamber, where they undergo quick evaporation by the hot air stream [6]. Usually, the inlet air temperature typically ranges from 150 °C to 220 °C, while the outlet air temperature is between 50 °C and 80 °C [6]. Differences in temperature and vapor pressure between the droplets and drying medium result in the evaporation of the solvent [3]. It is important to rapidly remove moisture while avoiding the degradation of heat-sensitive bioactives; relative humidity and drying kinetics also play a role in wall structure formation. The solvent exits the equipment in vapor form, and the remaining solute is collected in a container. The drying phase must balance the inlet and outlet temperatures.

### 2.4. Particle Collection

The dried powder is collected by the collection system, conventionally using the cyclone separator [3]. The centrifugal force is applied to powder particles inside the cyclone. In this stage, the design and efficiency of the cyclone separator or collection chamber affects the powder recovery rate, particle size uniformity, and yield.

## 3. Wall Materials Used for Microencapsulation by Spray-Drying

Choosing the appropriate wall material for spray-drying microencapsulation is an extremely important step, as the selected wall material influences encapsulation efficiency, mechanical stability, and the final shelf life of the encapsulated product [5]. The important characteristics that determine the suitability of the wall material are high solubility in aqueous solvents, good emulsifying properties, film-forming ability, as well as cost efficiency (Table 1).

Therefore, the combination of two or more wall materials is used more frequently for microencapsulation because no single encapsulating agent possesses all the desired properties [25]. According to Pudžiuvelytė et al.’s publication [26], using mixtures of two or more wall materials instead of one impacts the spray-dried powder’s moisture content, wettability, and flowability, as well as the encapsulation efficiency of the active ingredients. For example, by separately using sodium caseinate (20%) and beta-cyclodextrin (20–30%) as wall materials in liquid feed formulation, the obtained powder’s moisture was 6–8% when using different mixtures of sodium caseinate (0.5–1%), skim milk (6–10%), resistant maltodextrin (8–12%), and beta-cyclodextrin (0.5–1%), and the range of moisture was 3–5%. The range of wettability using only one wall material in the formulation varies from 40 to 348 s, and when using mixture of four wall materials, it varies from 62 to 105 s. The Carr index varied from 19.44 to 44.44% when using one encapsulating agent in the formulation, and when using a mixture of four agents, it varied from 24.44 to 37.14%. When using a single encapsulating agent (gum Arabic or resistant maltodextrin), the encapsulation efficiency of bioactive ingredients was very low, less than 20%, compared to the results obtained using a mixture of four wall materials, which reached 40–55% [26].

Both natural and synthetic polymers can be used in microencapsulation by spray-drying. Natural polymers are preferred over synthetic polymers since they are highly biocompatible and biodegradable. Biopolymers used for spray-drying microencapsulation are classified into polysaccharides, proteins, and lipids [5]. Natural polymers based on proteins and polysaccharides are the most commonly employed wall materials in spray-drying processes.

### 3.1. Polysaccharides

Polysaccharides are present in plants (e.g., cellulose and starch), animals (e.g., chitin), and microorganisms (e.g., dextran) [8]. These polymers are used as encapsulants for the following features: low costs, widespread availability, and stability. Examples of the most commonly used polysaccharides for spray-drying microencapsulation include maltodextrin, chitosan, and gum Arabic.

Maltodextrin is a partially hydrolyzed starch product that shows great solubility in water and protects encapsulated products from oxidation. It also has a neutral flavor and a relatively low viscosity at high solid concentrations. Maltodextrin is frequently used for hydro-soluble compounds such as anthocyanins [11]. However, maltodextrin does not have strong emulsifying properties due to the presence of hydroxyl groups that are responsible for wettability [18].

Chitosan is produced from chitin, which is naturally found in the outer skeleton of shrimps and crabs. Chitosan has a cationic nature because of amino groups that are responsible for its antibacterial, antifungal, and antiviral activity. It also has the ability to form gels. Chitosan is widely used as an encapsulant for many sensitive compounds including enzymes [27], fish oil, and curcumin [10]. The main drawback is its poor solubility in water [28]. Various derivatives of chitosan help to overcome this problem [28].

Gum Arabic is the most famous representative of gums. It is highly soluble in water and prevents the oxidative degradation of core materials. In addition, gum Arabic promotes good retention of volatile compounds (above 85%) during spray-drying. It is widely used as an encapsulating agent for lipo-soluble bioactive compounds [3]. However, problems related to its availability and cost limit its use.

### 3.2. Proteins

Milk proteins are used to encapsulate oil-soluble actives as they stabilize oil-in-water emulsions [15]. The major proteins found in milk are casein and whey.

Whey is derived from cheese-making processes [5]. Whey proteins are categorized into whey protein isolates or whey protein concentrates [5,15]. Additionally, wall compositions containing whey protein isolates, and whey protein concentrates provide remarkable protection against lipid oxidation.

Casein is obtained through the acidification of skimmed milk at a pH of 4.6 [5,15]. Sodium caseinate, the sodium salt of casein, has high solubility, low viscosity, and good emulsifying properties, making it suitable for microencapsulation. It has been reported that sodium caseinate effectively encapsulates orange oil and citric acid [15].

### 3.3. Innovative Wall Materials for Spray-Dying Microencapsulation

There is growing interest in substituting conventional wall materials (like maltodextrin and gum Arabic) with green wall materials for microencapsulation by spray-drying. The utilization of agro-industrial by-products, such as orange peels and brewery yeast, as raw materials for wall structures and drug delivery matrices, aligns with circular economy principles and offers measurable environmental benefits. In the European Union countries of Portugal, Spain, Italy, and Greece, orange production results in more than 6 million tonnes gathered each year. In Italy this production amounts to about 30%, with a corresponding generation of a voluminous waste stream (about 0.6 million tonnes of orange waste, containing approximately 50–60% *w*/*w* (wet weight) of the processed fruit, 60–65% *w*/*w* composed of peels, 30–35% *w*/*w* of internal tissue, and the remaining share of seeds) [29].

Orange peels are a rich source of sugars (30–40%), pectin (15–25%), cellulose (8–10%), and hemicellulose (5–7%). The soluble sugars can be directly utilized by *Gluconacetobacter xylinus* as a carbon source for bacterial cellulose (BC) production. Orange peels have great potential to be used as a feedstock in BC production, as the process is eco-friendly and promotes a circular economy. According to the studies, BC production showed up to six times higher yields than a conventional medium when orange peel waste was used as a carbon source [30].

Leftover orange peels after juice processing were used for the extraction of cellulose and for their conversion into cellulose nanocrystals (CNCs) for use in bionanocomposite films for packaging materials. The study shows the possibility of employing produced films as a green substitute to conventional plastic materials for packaging foods sensitive to microbiological decay and photo-oxidation. Furthermore, the extraction from agrifood waste and their application as nano-reinforcing agents minimize the environmental and economic costs related to waste disposal, thus creating a new opportunity for businesses with high growth potential [31].

Brewery yeast biomass or brewers’ spent grain when repurposed into value-added biocomponents avoids the generation of high-BOD wastewater, thereby mitigating environmental pollution and contributing to sustainable waste management. Life cycle assessment (LCA) studies have demonstrated that yeast-based SCPs produced from oat-processing side-streams show strong land-use benefits: they require ~61% less area than soy protein concentrates. According to the obtained data, the extraction of proteins from Brewers’ spent grain was shown to be the step with the greatest potential for advancing the technological maturity scale and has a smaller carbon footprint [32,33].

However, scaling up spray-drying processes using by-product-derived wall materials requires careful consideration. Variability in by-product composition that impacts quality, difficulty in maintaining process reproducibility at scale, and potential regulatory hurdles are key challenges that may hinder industrial applications.

### 3.4. Fibers

#### 3.4.1. Wood Hemicelluloses

Wood hemicelluloses, heterogenous polysaccharides obtained from the by-products of the forest industry, have recently gained attention as alternative encapsulating agents. Hemicelluloses have good surface activity, relatively low viscosity at high concentrations, and good stability. According to Thao et al., wood hemicelluloses have much higher oil encapsulation efficiency than gum Arabic [17]. In the study by Halahlah et al., spray-dried microparticles of bilberry juice prepared with wood-based hemicelluloses (namely galactoglucomannans and glucuronoxylans) demonstrated a relatively high encapsulation efficiency similar to that of gum Arabic. Moreover, wood hemicellulose-based powders exhibited significantly higher phenolic content and antioxidant activity than those of gum Arabic. The higher phenolic content values could be explained by the presence of lignan-derived phenolic compounds in the structure of galactoglucomannans and glucuronoxylans [34].

#### 3.4.2. Orange Waste Fiber

Kaderides et al. studied a new encapsulating agent from orange juice by-products as a wall material for spray-drying microencapsulation of phenolic compounds from pomegranate peel extract [19]. The study revealed that the encapsulation efficiency of pomegranate peel extract with orange waste fiber is higher than with common wall materials.

#### 3.4.3. Yeast

Yeast cells, by-products of fermentation processes, are considered as novel carrier materials of natural phytochemicals. The thick wall in all yeast species contains three major constituents: β-glucans polymers (glucose polysaccharides), mannan (mannose polysaccharide), and a small amount of chitosan [35]. Due to such a unique composition, yeast cell walls can protect the encapsulated substances from relatively high humidity, heat, light, or oxygen damage. Vélez-Erazo et al. improved the oxidative stability of sunflower oil using Saccharomyces cerevisiae as encapsulants [20]. In the study by Sultana et al., brewer’s yeast cells were successfully used to encapsulate flavors (d-limonene and ethyl hexanoate) by spray-drying [21].

#### 3.4.4. Canola Protein Isolate

Canola protein isolate is usually obtained from canola meal, which is a by-product of canola oil extraction. The major protein components found in canola meal are the storage proteins cruciferin and napin and the structural protein oleosin. Cruciferin (constituting 60–65% of the total protein) is a heat-stable protein with a high denaturation temperature (around 91 °C), and it is also gastro-resistant. In addition, it is regarded as a good emulsifier and gel-forming agent [36]. Such cruciferin properties have potential applications in the encapsulation of bioactive substances. The research by Wang et al. demonstrated that modifications of rapeseed protein isolate using high pressure improved its functional properties as wall materials for spray-drying microencapsulation of rapeseed peptides [37].

#### 3.4.5. Pea Protein Isolate

Pea protein isolate is extracted from peas (*Pisum sativum* L.). Pea protein isolates have good gelation and emulsifying properties. The limitations of pea protein isolates when used alone as wall materials are pH sensitivity and high viscosity at high concentrations. These problems can be solved by using pea proteins in combination with polysaccharides [24]. Several studies have been carried out using pea protein isolates to encapsulate curcumin [38], fish oil [39], flaxseed oil [40], and sunflower oil [41] via spray-drying. The study by Le Priol et al. showed that pea protein isolate is the most efficient wall material for sunflower oil protection compared to other plant proteins extracted from hemp, brown rice, soybean, and sunflower seeds. These results were directly related to the solubility and fraction composition of pea protein isolate. The good solubility and emulsifying properties of pea protein isolate were attributed to its main components, the 7S and 11S globulins [41].

## 4. Microencapsulated Substances

Many bioactive substances (e.g., polyunsaturated fatty acids) are frequently sensitive to light, temperature, oxygen, and other critical factors that consequently cause a loss of their bioactivity and limit their practical application in the pharmaceutical and food industries [42]. Spray-drying is a very common microencapsulation technique for various bioactive compounds, protecting materials from such environmental conditions [12,36]. Moreover, the spray-drying technology could be an alternative for masking the bitter or unpleasant taste of bioactive ingredients. Different bioactive components from plants, animal products, and other sources have been successfully spray-dried, resulting in stable preparations.

### 4.1. Phytochemicals

A broad spectrum of natural phytochemicals, including heat-sensitive phytochemical substances (e.g., polyphenols), could be converted into powder by this method with a low risk of degradation due to the rapid and short drying duration [11,36].

Polyphenols are the major group of phytochemicals that have multiple applications in the food and pharmaceutical industries. However, these compounds are often associated with limited water solubility, limited bioavailability, and an unpleasant taste [7]. To overcome such problems, spray-drying microencapsulation has been applied to various polyphenol-rich extracts (Table 2). In the study by Zanoni et al., microencapsulation by spray-drying demonstrated increased thermal stability of polyphenols from red chicory and red cabbage extracts without altering the color features of the pigments [43]. Another study by Cegledi et al. showed that spray-drying microencapsulation increased the bioavailability of polyphenols from nettle leaf extracts [44].

The spray-drying technique has also been employed for the microencapsulation of essential oils and complex mixtures of terpenes, terpenoids, and other aromatic constituents (Table 3). Factors limiting their commercial application possibilities are high volatility as well as sensitivity to external factors like heat and light [45]. The microencapsulation of essential oils by spray-drying is used to protect unstable essential oil components from evaporation and oxidation since they affect the flavor and functional properties of the essential oil. In the study by Bajac et al., spray-drying encapsulation has been successfully applied to protect the volatile compounds of juniper berry essential oil [45]. Carotenoids, known as lipophilic plant pigments, have been widely studied for their potential biological properties such as antioxidant and anti-inflammatory activities. Nevertheless, carotenoids are highly unstable and susceptible to processing and storage conditions, which restrict their use [46]. The spray-drying technique has been used to prevent such issues and preserve the bioactivity of carotenoids. In the study by Drosou et al., β-carotene was encapsulated by employing spray-drying and freeze-drying techniques. Spray-dried microparticles of β-carotene yielded higher encapsulation efficiency (85%) than freeze-dried ones (70%). Moreover, spray-dried encapsulants showed better protection of β-carotene against oxidative degradation induced by different storage temperatures and a_w_ levels, as well as UV-Vis irradiation [47]. According to González-Peña et al., spray-dried powders of mamey (*Pouteria sapota*) and carrot (*Daucus carota*) carotenoids possessed antioxidant activity even after three months of storage [46]. Carotenoids are poorly soluble in water due to their lipophilic nature. The spray-drying technology can enhance their solubility in water by utilizing water-based carriers. Lima et al. successfully spray-dried carotenoids from pumpkin peels using gum Arabic as an encapsulating agent. The produced powders had high water solubility, enabling their use in aqueous systems [48].

**Table 2:** Summary of wall materials, encapsulation efficiency, and functional benefits of encapsulated phenolic compound-rich extracts.

No.	Ingredient(s)	Wall Materials, Ratio	Processing Parameters	Encapsulation Efficiency, %	Benefits of Encapsulated Ingredients (Increase Solubility, Antioxidant Activity, etc.)	References
1.	Red cabbage (*Brassica oleracea*) anthocyanin-rich extract	Maltodextrin and Arabic gum, with citric acid. Different ratios were used; the best ratio was the one with the highest maltodextrin content: 25:25 (+1% of citric acid)	8 mL/min T_inlet_ = 130 ± 2 °C	The microparticles yield was higher than 40%.	The moisture content in microparticles varied between 6.25 and 16.42%, and the water activity ranged from 0.48 to 0.64, which is a positive value for future food application (not confirmed in other articles). The microparticles presented a uniform appearance and a homogenous size distribution, with spherical shapes and smooth surfaces.	[49]
2.	Barberry (*Berberis vulgaris*) extract, rich in anthocyanins	Combination of gum Arabic and maltodextrin (3:1 most effective), maltodextrin and gelatin (3:1), and only maltodextrin. The ratio between the extract solid content and the wall material was 1:4	T_inlet_ = 150 °C T_outlet_ = 100 °C	Efficiency: About 93% for Arabic gum with maltodextrin and 91% for others, according to the presented graph. Encapsulation efficiency of maltodextrin: Gelatin was significantly lower than maltodextrin/gum Arabic (*p* < 0.05).	Colorant has higher efficiency and the longest anthocyanin stability under all conditions evaluated. Formulating a jelly using the encapsulated anthocyanin color at the level of 7% was achieved with acceptable sensory attributes and physicochemical evaluations.	[50]
3.	Extracts of *Hibiscus sabdariffa* calyces with natural red–purple pigment and antioxidant properties	Different concentrations of mesquite gum (100:1–100:5 *v*/*w*)	T_inlet_ = 180 ± 2 °C T_outlet_ = 100 ± 2.3 °C	The highest yield was 74.9 ± 4.3% when there was 5% mesquite gum.	Powders solubilize easily in water or in aqueous ethanol. Good storage, physicochemical, and antioxidant properties (kept almost constant in different conditions at room T for 1 year).	[51]
4.	*Renealmia alpinia* (Rottb.) Maas fruit pericarp extract. Potential purple colorant	Maltodextrin, gum Arabic, and a 1:1 mixture of both	T_inlet_ = 150 ± 2 °C T_outlet_ = 98 ± 2 °C	Maltodextrin/Arabic gum yield (21.58%), only Arabic gum (19.47%), and Maltodextrin only (18.59%). Maltodextrin/gum Arabic coating significantly (*p* < 0.05) increased the yield when compared with gum Arabic and maltodextrin only microencapsulates.	The mixture with MDX:GA showed lower humidity content and the highest yield of powders. However, GA was better as an encapsulating material for the conservation of anthocyanins and phenolic compounds; MDX showed superior coating capacity in encapsulates stored at 4 °C. It improved and preserved storage and antioxidant properties.	[52]
5.	Pomegranate juice powder	Maltodextrin (25, 35, and 45% *w*/*w*)	0.5–1.5 kg/h T_inlet_ 123–143 °C T_outlet_ 48–76 °C	The production yield of pomegranate juice powder was between 17 and 25%, with higher maltodextrin rates and temperatures leading to higher yields.	Affected density, anthocyanin level, and antioxidant properties; produced larger particles; and improved stability.	[53]
6.	Antioxidant-rich blueberry waste extracts	Sodium alginate (3% *w*/*w*) or inulin (3% *w*/*w*)	12 mL/min T_inlet_ = 150 ± 1 °C T_outlet_ = 80 ± 2 °C	The yields of “BWM-Alginate” and “BWM-Inulin” powders were 64∼72 and 60~68%, respectively.	The product generated from BWM had desirable color, water activity, and reconstitution properties in water or milk and can be used as a food colorant and supplement. Compared with inulin, alginate gave a greater powder yield, greater Bifidobacterium-boosting effects, better protection of antioxidants during spray-drying, and prolonged storage at 20 or 38 °C.	[54]
7.	Cabernet Sauvignon and Bordeaux grape pomace extracts	Brewery waste yeast Saccharomyces cerevisiae (5% (*w*/*w*) of dry yeast)	T_inlet_ = 130 °C T_outlet_ = 80 °C		Yeasts were proven to be a great wall material for the encapsulation of bioactive compounds by spray-drying. It was possible to obtain powders with characteristics that enhance the shelf-life of the product (1 year). Bordeaux grape pomace extracts are better as a colorant.	[55]
8.	Elderberry (*Sambucus nigra*) extract	Maltodextrin–β-glucan (0.5, 1, 2, and 3%) and control/maltodextrin and Arabic gum (92.5:7.5)	The flow of the pump was 25%. T_inlet_ = 140 °C	3% BG—77.97% ± 2.350; control sample—80.45% ± 1.3855. The highest encapsulation efficiency was achieved with the powder containing the lowest ratio of maltodextrin/β-glucan, reaching around 93.9% ± 2.717 compared to other maltodextrin/β-glucan ratios tested (*p* < 0.05).	The 0.5% β-glucan ratio is recommended for more efficient microencapsulation due to the encapsulation efficiency, storage loss, ascorbic acid, and anthocyanin total content characteristics. However, while the higher content of maltodextrin or Arabic gum is undesirable, the higher β-glucan content as its replacement is favorable due to its health-beneficial effect on the human body.	[56]
9.	Blue maize (*Zea mays*) polyphenols	Maltodextrin (120 g/L total solids) or maltodextrin/pectin (120 g/L total solids, 84/16 *w*/*w*)	T_inlet_ = 150 ± 1 °C T_outlet_ = 80 ± 1 °C	80.61 ± 1.32 for maltodextrin and 65.76 ± 0.19 for the maltodextrin–pectin combination. The maltodextrin particles had a higher yield, as compared to maltodextrin–pectin particles (*p* < 0.05).	The combined matrix showed better protection during storage, with a significantly higher half-life and antioxidant activity. The release of phenolics after in vitro digestion was nearly complete from both matrices; the combined matrix favored intestinal release but less absorption.	[57]
10.	Anthocyanins from chokeberry (*Aronia melanocarpa)*	92.5% maltodextrin and 7.5% guar gum, gum Arabic, pectin, β-glucan, or inulin	The flow of the pump was 25%. T_inlet_ = 140 °C	Efficiency varied from 78.61% for Maltodextrin + Arabic gum to 92.98% for Maltodextrin + guar gum. GG showed the highest efficiency of encapsulation. The lowest efficiency was obtained with microcapsules coated by maltodextrin and gum Arabic compared to the other groups (*p* < 0.05).	The particles of the GG powder were the smallest, preferably distributed, and of uniform size and shape. These features directly influenced the highest solubility of this preparation among all powders. Guar gum powder also had the best protecting properties.	[58]

### 4.2. Oils

Vegetable and marine oils are defined as functional foods rich in unsaturated fatty acids. Unfortunately, oils with high amounts of polyunsaturated fatty acids are prone to oxidative damage, resulting in the development of an unpleasant taste and odor, as well as color and viscosity reversion. Microencapsulation is considered to be an effective method to protect such oils. Many polyunsaturated fatty acid-rich oils have been microencapsulated using the spray-drying technique, such as flaxseed, safflower, and fish oils [59,60]. In the study by Zhang et al., flaxseed oil and safflower seed oil powders were successfully produced by spray-drying and achieved high encapsulation efficiency (99%). Moreover, the oil powders remained stable over six months under 30 °C and 60% relative humidity. The study also showed that the oral supplementation of the spray-dried flaxseed oil powder significantly ameliorated inflammatory responses in mice with DSS-induced colitis compared with lipid flaxseed oil [59]. Essential oils (EOs), although highly valuable for their antimicrobial, antioxidant, anti-inflammatory, and fragrance properties, are very sensitive to environmental factors. Therefore, their microencapsulation is important to preserve their stability and efficacy. EOs have also been effectively microencapsulated using spray-drying to preserve their volatility and bioactivity [61].

For instance, citrus essential oils (orange, lemon, and grapefruit) incorporated into microencapsulated fish oil matrices resulted in lower peroxide values (13–14 meq O_2_/kg) compared to controls (~25 meq O_2_/kg) after storage, indicating improved oxidative protection [62].

The spray-drying technique for EOs enhances thermal and oxidative stability, preserves biological activities, enables controlled release, and modifies organoleptic properties (Table 2).

**Table 3:** Summary of wall materials, encapsulation efficiency, and functional benefits of encapsulated essential oils.

No.	Ingredient(s)	Wall Materials, Ratio	Processing Parameters	Encapsulation Efficiency, %	Benefits of Encapsulated Ingredients (Increase Solubility, Antioxidant Activity, etc.)	References
1.	Citronella (*Cymbopogon winterianus*) essential oil	Gum Arabic, maltodextrin (40%), and whey protein concentrate (60%)	T_inlet_ = 120 °C T_outlet_ = 65–70 °C	53–100%	Enhanced thermal stability, improved oxidative stability, and enabled controlled release	[63]
2.	Persian lime (*Citrus latifolia*) essential oil	Maltodextrin (20–35%)	120–300 mL/h T_inlet_ 120–180 °C	56–87% The encapsulation efficiency exhibited an increasing trend with the elevation of the maltodextrin concentration from 20% to 30% (*p* < 0.05).	Improved antimicrobial and thermal stability and the release of active substances	[64]
3.	Cardamom (*Elletaria cardamomum*) essential oil	Skim milk (10, 20, and 30%); modified starch (10, 20, and 30%)	10 mL/min T_inlet_ = 180 ± 10 °C T_outlet_ = 90 ± 10 °C	79–95%	Modified the size of particles and improved the release of active substances	[65]
4.	Cinnamon essential oil	Maltodextrin (15.75%) and whey protein isolate (7%)	T_inlet_ = 180 °C T_outlet_ = 90 °C	84–89% The encapsulation efficiency increased with decreasing whey protein isolate/maltodextrin ratios (*p* < 0.05).	Demonstrated strong protection with the optimized formulation during storage	[66]
5.	Pomelo (*Citrus grandis* (L.) *Osbeck*) essential oil	Maltodextrin (20–35%)	120 mL/h T_inlet_ = 140 °C	89.44% The effect of maltodextrin concentrations on encapsulation efficiency was statistically significant (*p* < 0.05).	Improved thermal stability and higher amount of components	[67]
6.	Corn mint (*Mentha arvensis* L.) essential oil	Maltodextrin (20–30%)	4–10 mL/min T_inlet_ 130–150 °C	68.6–98.9% Encapsulation efficiency increased with increasing maltodextrin concentrations (*p* < 0.05).	Higher amount of components	[68]
7.	Lavender essential oil	Gum acacia, sodium caseinate, gelatin, chitosan, β-cyclodextrin, and polyvinyl alcohol (1:1)	T_inlet_ = 180 °C T_outlet_ = 80 ± 2 °C	20–65% The encapsulation efficiency of lavender oil significantly improved when gelatin was used as a carrier material during spray-drying with gum acacia and sodium caseinate, increasing from 28.6% to 65.9% (*p* < 0.05).	Combinations of different wall materials would change the retention and release of essential oil and modify the surface morphology of microcapsules	[69]
8.	Ginger (*Zingiber officinale*) essential oil	Gum Arabic (20%) and inulin (20%)	0.8 L/h T_inlet_ = 160 °C T_outlet_ = 68 °C	9.46–35.69%	Improving the retention of bioactive compounds	[70]
9.	Basil (*Ocimum basilicum* L.) essential oil	Sodium alginate with sodium caseinate and maltodextrin (1:2 and 1:1)	T_inlet_ = 140 °C T_outlet_ = 70 °C	43–78% The encapsulation efficiency increased with increasing concentrations of sodium alginate in the mixture with maltodextrin (*p* < 0.05).	Increases solubility and release and improves encapsulation efficiency and morphological characteristics	[71]
10.	Clove (*Syzigium aromaticum*) essential oil	Casein (4.65%)	T_inlet_ 110–120 °C T_outlet_ 55–65 °C	97.78%	Improves encapsulation efficiency and antibacterial activity	[72]

### 4.3. Vitamins

Vitamins exhibit a broad range of biological activities, but their practical application is often limited due to high instability and sensitivity to external factors including oxidation, heat, and light. In order to avoid degradation and the loss of vitamin activity, the spray-drying technique has been used for preserving vitamins (Table 4). By applying this encapsulation technology, it is also possible to achieve the controlled release of vitamins over time and mask their organoleptic characteristics. Many studies have demonstrated the successful application of the spray-drying technique in the encapsulation of both water- and fat-soluble vitamins.

#### 4.3.1. Water-Soluble Vitamins

Chatterjee et al. successfully encapsulated vitamin B1 and vitamin B6 in spherical microspheres composed of ferulic acid-grafted chitosan by using the spray-drying process. Furthermore, vitamin B1- and vitamin B6-loaded ferulic acid-grafted chitosan microparticles showed anti-inflammatory activity in a rat paw edema model [73]. Barra et al. demonstrated that vitamin C can be successfully protected from oxidation by spray-drying. The results showed that microencapsulation significantly increased the thermal stability of vitamin C (up to 188 °C) when using sodium alginate and gum Arabic as encapsulating agents [74]. Estevinho et al. reported the efficient encapsulation of vitamin B9 (around 100%, except for formulations of vitamin C with modified starch) by the spray-drying method with different wall materials. The results showed a product yield between 13.1 and 49.8% [75].

#### 4.3.2. Fat-Soluble Vitamins

Ribeiro et al. demonstrated the possibility of encapsulating vitamin E using different wall materials by the spray-drying technique. The results indicated that the release kinetics of vitamin E were closely related to the wall materials. Formulations of vitamin E with inulin, maltodextrin, and sodium alginate demonstrated the fast-release characteristics of vitamin E, while gum Arabic, modified chitosan, starch, and modified starch slowed down the release of vitamin E [76]. In the study by Gonçalves et al., vitamin A–gum Arabic composite particles (15 and 20% (*w*/*v*) of gum Arabic) produced by the spray-drying technology remained stable after one month of storage [77]. Bashir et al. reported that the spray-dried powder of vitamin D presented better release behavior features compared to freeze-dried encapsulates. In addition, the spray-drying technique showed higher encapsulation efficiency of vitamin D3 than the freeze-drying technique [78].

**Table 4:** Summary of wall materials, encapsulation efficiency, and functional benefits of encapsulated vitamins.

No.	Ingredient(s)	Wall Materials	Processing Parameters	Encapsulation Efficiency, %	Benefits of Encapsulated Ingredients (Increase Solubility, Antioxidant Activity, etc.)	References
1.	Vitamin D3	Maltodextrin (25%), modified starch (3%), and whey protein (2%)	T_inlet_ = 170 °C T_outlet_ = ±80 °C	Maltodextrin/modified starch/whey protein yield 96.4%	Improved stability and bioavailability. An increase in maltodextrin concentration in the feed solution led to the formation of smoother and more spherical spray-dried powder particles.	[78]
2.	Vitamin E	Whey protein (1:3)	4 mL/min T_inlet_ = 100 °C T_outlet_ = 80 °C	89.6 ± 2.5%	Improved stability and bioavailability.	[16]
3.	Vitamin A	Gum Arabic, maltodextrin, and starch (15%)	4 mL/min T_inlet_ = 150 °C T_outlet_ = 80 °C	88–98%	Improved stability and bioavailability, extending the release time of vitamin A.	[79]
4.	Vitamin B1	Chitosan and ferulic acid (1:1)	10 mL/min T_inlet_ = 140 °C T_outlet_ = 77 °C	91 ± 2.31%	Vitamin B1-loaded microspheres showed potential anti-inflammatory activity via the inhibition of carrageenan-induced paw edema in albino rats.	[73]
5.	Vitamin B6	Chitosan and ferulic acid (1:1)	10 mL/min T_inlet_ = 140 °C T_outlet_ = 77 °C	83 ± 3.17%	Vitamin B6-loaded microspheres showed potential anti-inflammatory activity via the inhibition of carrageenan-induced paw edema in albino rats.	[73]
6.	Folic acid	Maltodextrin (40%)	1.5 L/h T_inlet_ = 194.2 °C T_outlet_ = 87.7 °C	90.9 ± 1.8%	Improved stability and bioavailability.	[80]
7.	Vitamin B12	Zein (20%)	4 mL/min T_inlet_ = 90 °C T_outlet_ ± 50 °C	82.3%	Improved stability, bioavailability, and release profiles of vitamin B12.	[81]
8.	Vitamin C	Sodium alginate (3.5%)	T_inlet_ = 110 °C T_outlet_ = 65 °C	93.48%	Alginate-based microparticles acted as a protective barrier, effectively preventing vitamin C from degrading or being lost during the 30-day storage period. After 30 days of storage, there was no statistically significant difference in encapsulation efficiency (*p* < 0.05).	[82]

### 4.4. Controlled Release Systems

Spray drying enables the development of controlled-release formulations that can improve the delivery of nutrients or bioactive compounds, especially in the pharmaceutical and food industries. Spray-dried microparticles can be designed to release their contents in a controlled manner to specific target sites by responding to specific stimuli such as changes in pH and temperature or the presence of specific enzymes. pH-induced release has been widely employed in designing drug delivery systems. For example, Gonciarz et al. developed spray-dried pH-sensitive microparticles of chitosan loaded with *M. bovis* BCG for the supporting treatment of *H. pylori* infection. These microparticles released live mycobacteria at a pH of 3.0 (when coated with N-Acetyl-d-Glucosamine) or a pH of 8.0 (when coated with Pluronic F-127) [83]. Medeiros et al. designed temperature- and pH-sensitive poly(N-vinylcaprolactam)-based spray-dried microparticles for the controlled release of ketoprofen. The in vitro drug release profile of ketoprofen at different temperatures and pH values indicated that poly(N-vinylcaprolactam) could be a promising carrier for pH- and temperature-responsive drug delivery systems [84].

Spray drying has been applied to achieve enzyme-triggered controlled release of encapsulated materials. In the study by Tallian et al., the spray-drying technique has been utilized to generate lysosome-responsive spray-dried chitosan microparticles for visual lysozyme detection in wound fluids. Spray-dried N-acetyl chitosan particles showed a five times faster lysozyme-mediated release of dyed chitosan fragments compared to non-spray-dried N-acetylated chitosan flakes [85].

## 5. Limitations and Future Directions

In spray-drying, researchers utilize various combinations of wall materials to achieve specific encapsulation properties (e.g., encapsulation efficiency, particle size, and stability), with different measurement methods and analysis criteria used to evaluate their performance. However, varying pairings of wall materials as well as analysis methods across studies can lead to conflicting conclusions and make it difficult to compare the findings. Many studies on spray-dried microcapsules utilize in vitro testing. Nevertheless, in vitro studies may not accurately reflect the potential effects on humans. Additionally, there is a lack of data on the long-term storage and stability of spray-dried microparticles, as many studies report only short-term storage outcomes.

Combining natural and synthetic wall materials in spray-drying may result in favorable characteristics of spray-dried microcapsules (e.g., enhanced stability and bioavailability of the final product) and may also potentially make the process more economical. Designing stimuli-responsive release systems (such as those triggered by pH, enzymes, or temperature) for spray-dried microcapsules offers a promising strategy for the targeted delivery of active compounds. More data is needed on the behavior of spray-dried microcapsules over extended periods or within complex matrix systems (e.g., food or cosmetics) for developing effective and shelf-stable formulations. Spray-drying could be applied in personalized delivery formulations, especially in the pharmaceutical and food industries, in order to meet specific patient needs or consumer preferences.

## 6. Conclusions

Microencapsulation by spray-drying is one of the most versatile and widely used technologies for the protection and preservation of sensitive bioactive compounds. This technology allows for the effective stabilization of components such as polyphenols, essential fatty acids, vitamins, and essential oils, protecting them from negative environmental influences—light, oxygen, humidity, and temperature changes.

This article reviews the main stages of the spray drying process and discusses the most commonly used wall materials, as well as their properties, advantages, and disadvantages. It also presents innovative encapsulation materials obtained from renewable sources, which help to increase the sustainability of the microencapsulation process. It has been proven that by using both traditional and new carrier systems, it is possible to achieve high encapsulation efficiency, improve the stability of bioactive substances, and control their release.

In the future, much attention will be paid to the development of stimulated (e.g., pH- and temperature-based) release systems for active ingredients and personalized microencapsulation solutions that allow for the tailoring of formulations to individual needs. The sustainability aspect—the search for new wall materials from waste or by-products—will also remain a very important trend. However, several research gaps remain at the moment. There is a clear need for standardized testing protocols and methodological harmonization in assessing release profiles, encapsulation efficiency, and stability over time. In addition, the development of hybrid wall materials that combine natural and synthetic polymers could offer a balance between biodegradability, mechanical strength, and processability. These directions may help overcome current limitations and improve the reproducibility and scalability of microencapsulation systems.

Microencapsulation by spray-drying will continue to be one of the key methods to preserve, improve, and more effectively utilize sensitive bioactive compounds in various industrial fields.

## Figures and Tables

**Table 1:** Comparative characteristics of wall materials used in spray-drying microencapsulation.

Wall Material	Type	Water Solubility	Emulsifying Capacity	Film-Forming Ability	Encapsulation Efficiency	Cost	References
Maltodextrin	Polysaccharide	High	Low	Good	Moderate–High	Low	[11]
Gum Arabic	Polysaccharide	Very High	High	Excellent	High	Medium–High	[12]
Chitosan	Polysaccharide	Low–Moderate	Moderate	Good	High	Medium	[13,14]
Whey Protein Isolate	Protein	High	High	Good	High	Medium	[15,16]
Sodium Caseinate	Protein	High	High	Good	High	Medium	[15]
Wood Hemicelluloses	Polysaccharide	Moderate–High	Good	Moderate	High	Low	[17,18]
Orange Waste Fiber	Fiber	Moderate	Low	Moderate	High	Very Low	[19]
Yeast Cell Wall	β-glucan composite	Low–Moderate	Moderate–High	High	High	Very Low	[20,21]
Canola Protein Isolate	Protein	Moderate	Good	Good	High	Low	[22,23]
Pea Protein Isolate	Protein	Moderate	Moderate	Good	Moderate–High	Low	[24]

## Data Availability

Data is contained in the paper.
